# Correction: Optical humidity sensors based on lead-free Cu-based perovskite nanomaterials

**DOI:** 10.1039/d2na90084j

**Published:** 2022-11-01

**Authors:** Hoseok Lee, Donghwa Lee, Haedam Jin, Dohun Baek, Mi Kyong Kim, Jeongbeom Cha, Sung-Kon Kim, Min Kim

**Affiliations:** School of Chemical Engineering, Jeonbuk National University Jeonju 54896 Republic of Korea skkim@jbnu.ac.kr; Graduate School of Integrated Energy-AI, Jeonbuk National University Jeonju 54896 Republic of Korea minkim@jbnu.ac.kr

## Abstract

Correction for ‘Optical humidity sensors based on lead-free Cu-based perovskite nanomaterials’ by Hoseok Lee *et al.*, *Nanoscale Adv.*, 2022, **4**, 3309–3317, https://doi.org/10.1039/D2NA00168C.

The authors regret that the funding information was incorrectly shown in the acknowledgements section of the original manuscript. The authors wish to replace the grant number, “NRF-2021M2D2A1A0204148211”, with “NRF-2021M2D2A1A02041482”. The corrected funding acknowledgement is as shown below.

S.-K. K. acknowledges the support from the National Research Foundation of Korea (NRF) grant funded by the Korea government (MSIT) (NRF-2021M2D2A1A02041482).

The Royal Society of Chemistry apologises for these errors and any consequent inconvenience to authors and readers.

## Supplementary Material

